# Conservative Treatment of Complicated Crown Fractures Utilizing Mineral Trioxide Aggregate and Adhesive Reattachment of Tooth Fragments: A Case Series

**DOI:** 10.7759/cureus.109186

**Published:** 2026-05-19

**Authors:** Sanjana Jayakumar Nair, Jinesh A, Saurabh Gupta, Harsh Chansoria, Gopika Krishnan K G

**Affiliations:** 1 Department of Conservative Dentistry and Endodontics, Government College of Dentistry, Indore, IND; 2 Department of Prosthodontics, Government College of Dentistry, Indore, IND

**Keywords:** adhesive reattachment, complicated crown fracture, conservative treatment, dental trauma, mineral trioxide aggregate (mta), pulp capping, pulpotomy, pulp vitality, tooth fragment

## Abstract

This case series highlights the conservative management of complicated crown fractures in permanent incisors using mineral trioxide aggregate (MTA) for pulp capping and adhesive techniques for fragment reattachment. Three patients aged 15-30 were treated and monitored over 12 months. All cases showed positive outcomes with maintained pulp vitality and satisfactory aesthetic and functional results. No signs of periapical pathology or secondary caries were observed. The adhesive reattachment of the tooth fragments provided excellent marginal adaptation and colour match, enhancing the overall success of the treatment.

## Introduction

A crown fracture is the most prevalent outcome of traumatic injuries, primarily impacting the permanent teeth. There is no preference for either side, but the area of the maxillary central incisor is most commonly affected [1]. In a study in Turkey, it was seen that the percentage of dental trauma was much greater in male adolescents than in female adolescents; 17.41% of male and 7.9% of female adolescents were affected, with a male-to-female ratio of 2.18 [2]. 

Complex crown fractures occur in a range of 0.9-13% of all dental injuries [3]. According to a study by Canakci et al., falls account for 27.7% of trauma-related incidents. Subsequently, violence and conflict have emerged as the second most prevalent source of trauma, accounting for 24% of cases, closely followed by sports-related injuries at 18.8%. The incidence of trauma caused by collisions and traffic accidents accounts for 13.7% and 11.7% of all cases, respectively. The remaining causes account for 4.5% [2]. In central India, the reported prevalence of traumatic dental injuries of permanent incisors is 12.8% [4].

The degree of pulp exposure may vary from a minute pinpoint exposure to total exposure of the coronal pulp. The exposure of the pulp makes the treatment challenging. It has been observed that if left exposed, the pulp might become necrotic through bacterial contamination. The outcome of treatment depends on the extent of the injury, the quality and timeliness of the initial care, and the recall protocol [1,5]. The maintenance of pulp vitality using existing vital pulp treatment (VPT) interventions is an essential element in the management of injured teeth. Multiple VPT interventions have been extensively documented, and their objective is to preserve the vitality of the pulp tissue. Partial (Cvek) pulpotomy entails removing a portion of the coronal pulp in order to achieve hemostasis and then placing a pulp capping material [6]. The clinical signs and symptoms used to evaluate the preoperative pulpal status do have some correlation with the histologic pulpal diagnosis [7]. There are studies that have demonstrated significant clinical success when mineral trioxide aggregate (MTA) is employed in vital pulp therapy [8,9]. MTA is an alkaline substance that promotes the formation of dentin bridges and possesses excellent sealing properties and biocompatibility. Additionally, it exhibits a high level of adhesion to composite materials. It exhibits radiopacity [10].

This report of three cases aims to assess the outcome of MTA in performing a partial pulpotomy and subsequently reattaching a tooth fragment using resin composite. For standardization of clinical assessment, all cases were evaluated using predefined criteria. The size of pulp exposure was clinically estimated using a periodontal probe and categorized as small (<1 mm), moderate (1-2 mm), or large (>2 mm). The time interval between trauma and treatment was recorded in hours based on the patients' history. Pulp vitality was assessed using electric pulp testing at baseline and during follow-up visits.

## Case presentation

Case 1

A 15-year-old patient presented at the Department of Conservative Dentistry and Endodontics with two fractured upper anterior teeth. The patient reported falling from a bicycle 10 hours ago. During the clinical examination, a fracture of Ellis Class III was observed, with pulp involvement in teeth 11 and 21. Under stimulus, the patient felt pain.

The visible pulp exhibited a bright red color, signifying a well-established blood flow to the pulp. While the pulp vitality tests were non-affirmative, the teeth demonstrated normal responses to both electric pulp testing and thermal testing conducted on the labial surface, which was suggestive of pulp in good health. Furthermore, there was a complete absence of mobility. Two treatment options were proposed after analyzing the clinical conditions and additional tests. The first option was conventional root canal therapy, and the second was a partial pulpotomy in teeth 11 and 21, which would have preserved the vitality of these teeth. The patient's preference was to proceed with a partial pulpotomy.

The teeth was anaesthetized with local infiltration of 0.6 ml lignocaine (1:200000 adrenaline) and isolated with a rubber dam. Chlorhexidine was used to disinfect the teeth. A high-speed diamond bur with water coolant was used to excise the superficial layer of the exposed pulp and the surrounding tissue to a depth of 2 mm. To control bleeding, the first step was to irrigate the affected area with isotonic saline solution. Next, a cotton pellet soaked in 2.5% sodium hypochlorite was applied for two minutes. This helped to stop the bleeding. Finally, the area was irrigated once again with isotonic saline solution. MTA Angelus Powder (Angelus Odontologia, Londrina, Brazil) was combined with distilled water in a 3:1 ratio to achieve a wet, gel-like consistency. The MTA carrier was utilized for delivering the prepared MTA, while pluggers were employed for the purpose of condensation. The setting time was 15 minutes.

The teeth and fragments were subjected to selective enamel etching using 37% phosphoric acid (Eco-Etch; Ivoclar Vivadent AG Schaan, Liechtenstein). Following this, the GC SOLARE Universal Bond adhesive system (GC International AG, Lucerne, Switzerland) was applied, and the GC SOLARE Sculpt (GC International AG) in A2 shade was applied as an intermediate layer to bond the fragment onto the teeth. Next, the material was light-cured on the buccal and palatal sides of the teeth for a duration of 20 seconds each. The teeth underwent initial finishing using fine and extra-fine diamond burs and were polished using either a coarse, medium, fine, or super-fine grit Sof-Lex disc (3M Company, Maplewood, Minnesota, United States). Figures 1-8 show a step-by-step procedure.

**Figure 1:**
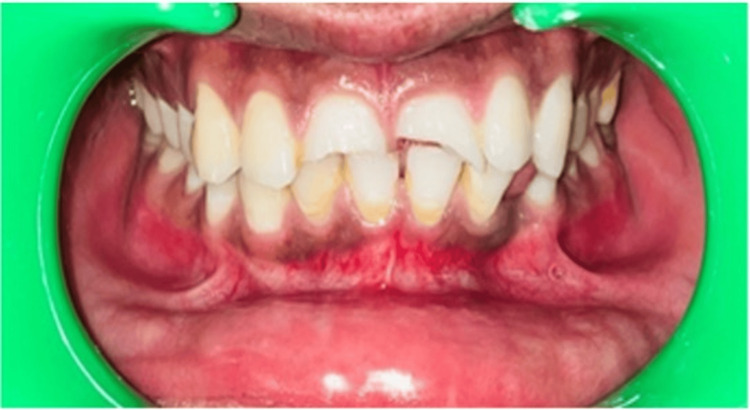
Preoperative image from buccal view showing Ellis class III fracture of 11 and 21 (Case 1)

**Figure 2:**
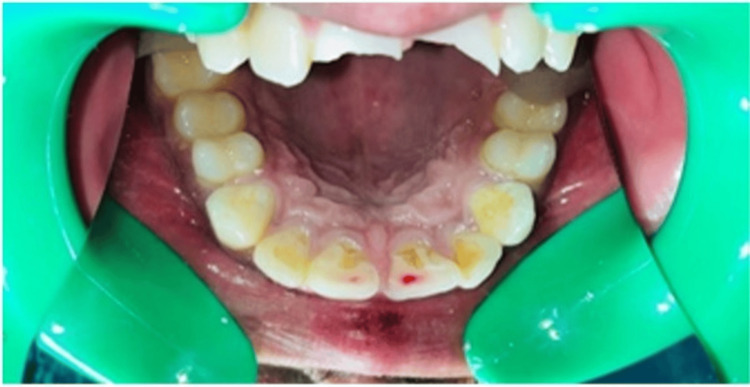
Preoperative image from palatal view showing pulp exposure of 11 and 21 (Case 1)

**Figure 3:**
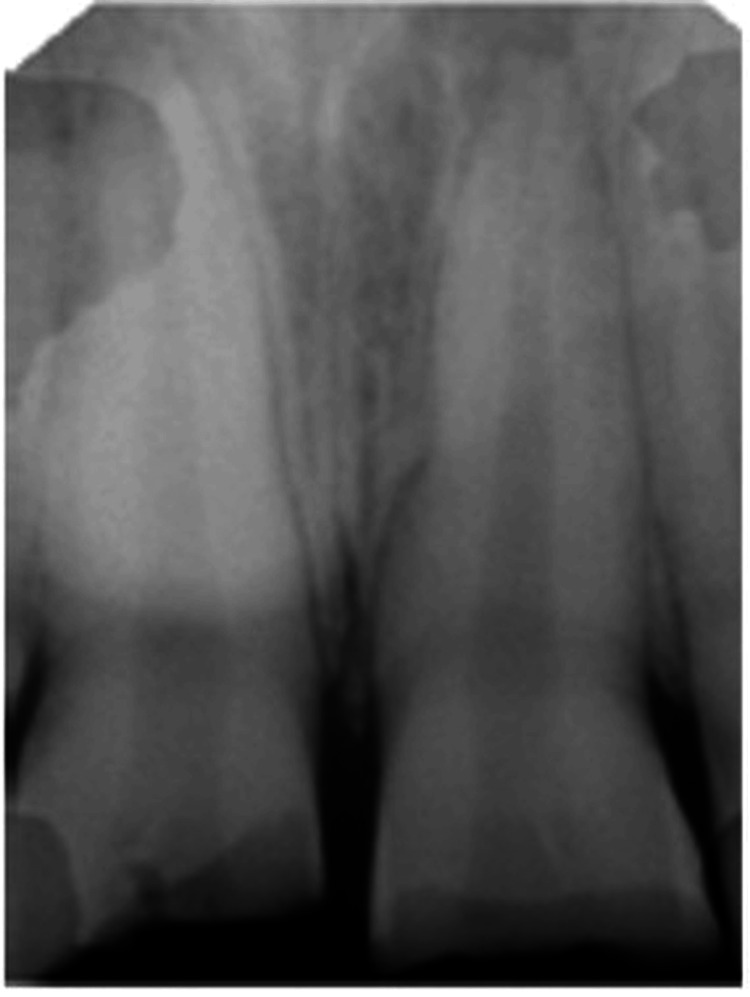
Preoperative radiograph of 11 and 21 (Case 1)

**Figure 4:**
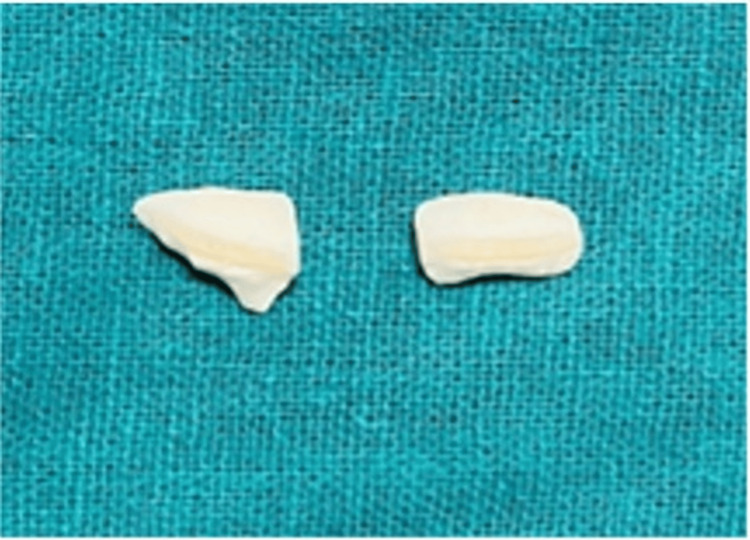
Tooth fragments of 11 and 21 (Case 1)

**Figure 5:**
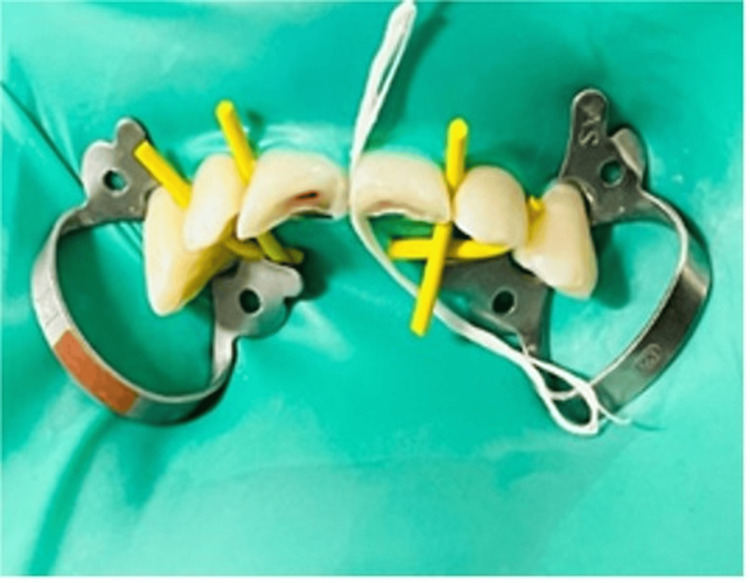
Isolation using rubber dam (Case 1)

**Figure 6:**
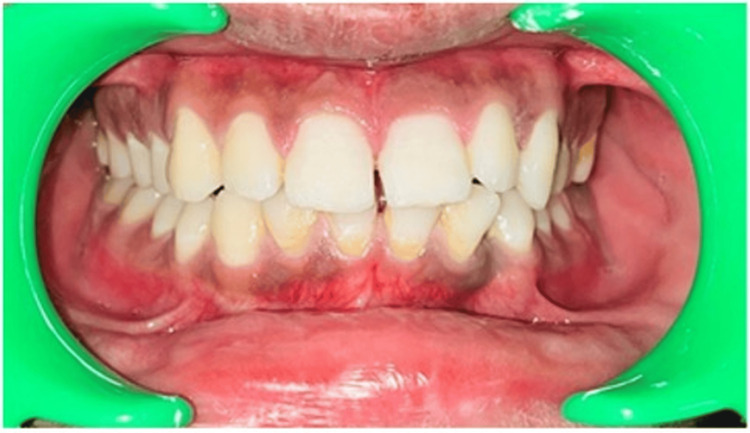
Immediate postoperative image (Case 1)

**Figure 7:**
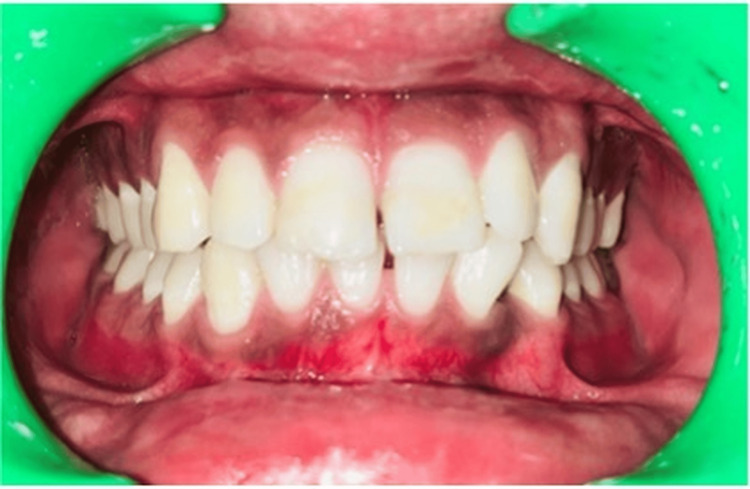
Postoperative image after 12 months (Case 1)

**Figure 8:**
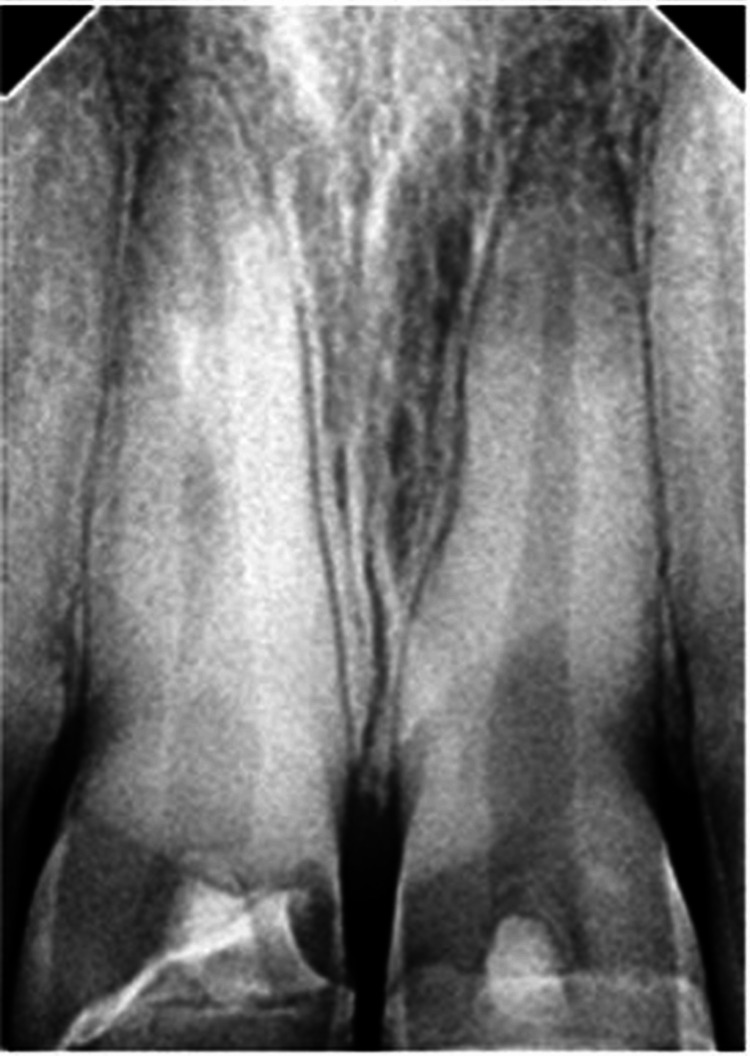
Postoperative radiograph after 12 months showing reparative dentin formation just apical to MTA (Case 1) MTA: mineral trioxide aggregate

Case 2

A 29-year-old patient presented at the Department of Conservative Dentistry and Endodontics with a case of fractured upper anterior teeth. During the clinical examination, a fracture of Ellis Class III was observed, with pulp involvement in tooth 11. The fragment was stored in an isotonic saline solution by the patient until they reported to the department the following day after the fall injury. Under stimulus, the patient felt pain.

The pulp, which was visible, was a bright red colour, indicating that blood was flowing to it adequately. The pulp vitality tests, similar to Case 1, showed normal responses to both electric pulp testing and thermal testing on the labial surface, suggesting that the pulp was in good health. Mobility was absent, and after reviewing the patient's medical history, two treatment options were suggested. The first was conventional root canal therapy, while the second was to preserve tooth 11 by performing a partial pulpotomy followed by fragment reattachment using composite resin. Per the patient's wishes, a partial pulpotomy was performed. Figures 9-17 show a step-by-step procedure.

**Figure 9:**
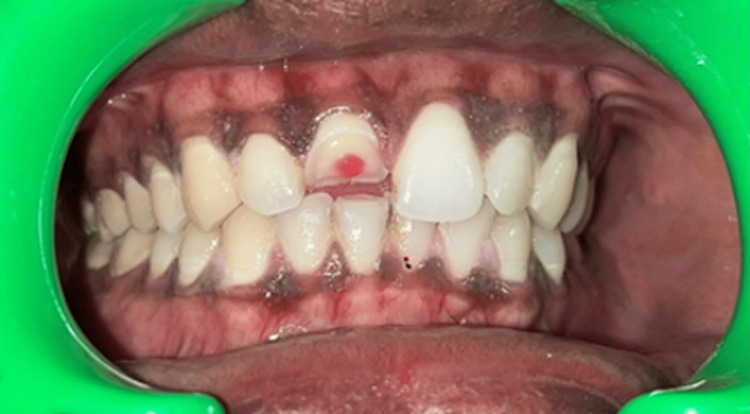
Preoperative image from buccal view showing Ellis class III fracture of 11 (Case 2)

**Figure 10:**
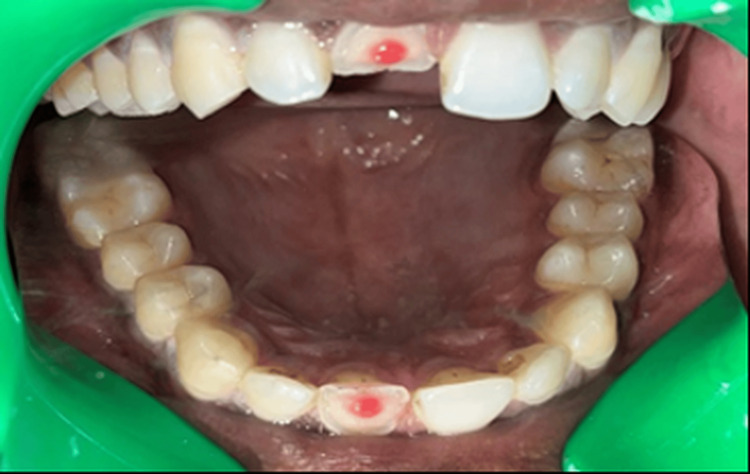
Preoperative image from palatal view showing pulp exposure with 11 (Case 2)

**Figure 11:**
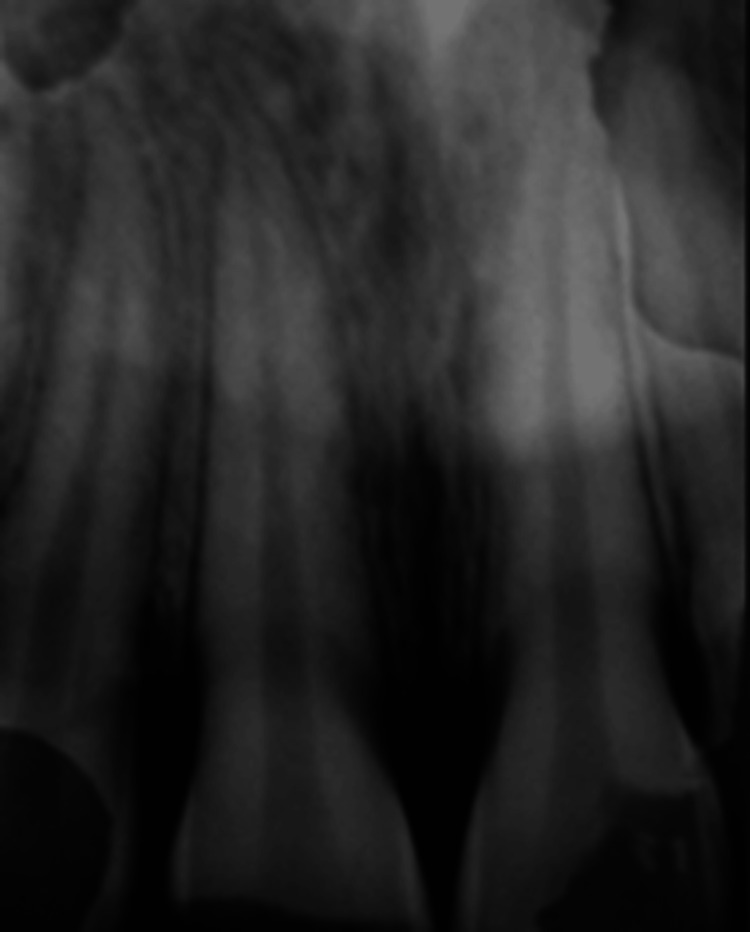
Preoperative radiograph of 11 (Case 2)

**Figure 12:**
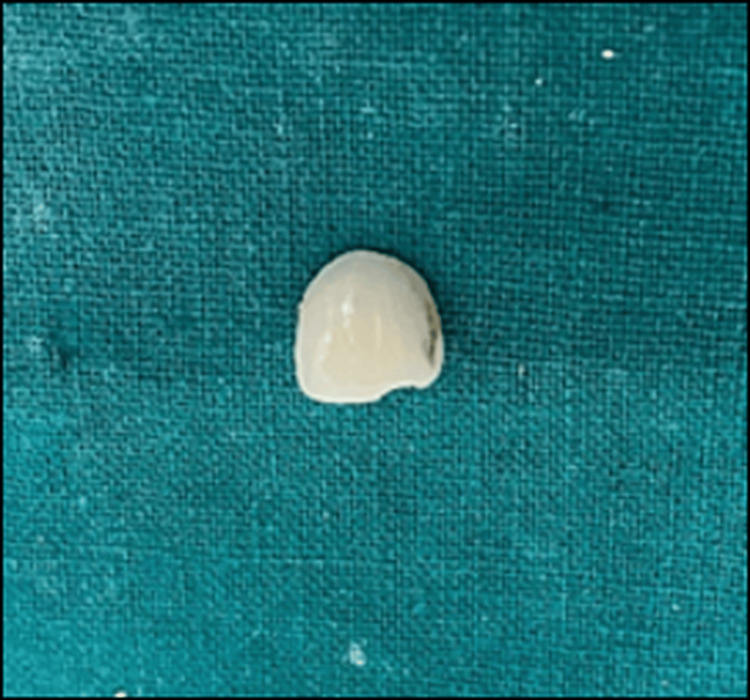
Tooth fragment of 11 (Case 2)

**Figure 13:**
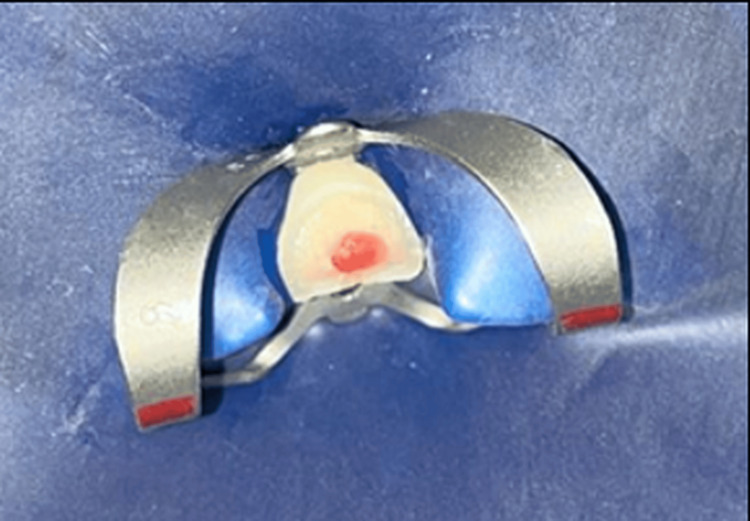
Isolation using rubber dam (Case 2)

**Figure 14:**
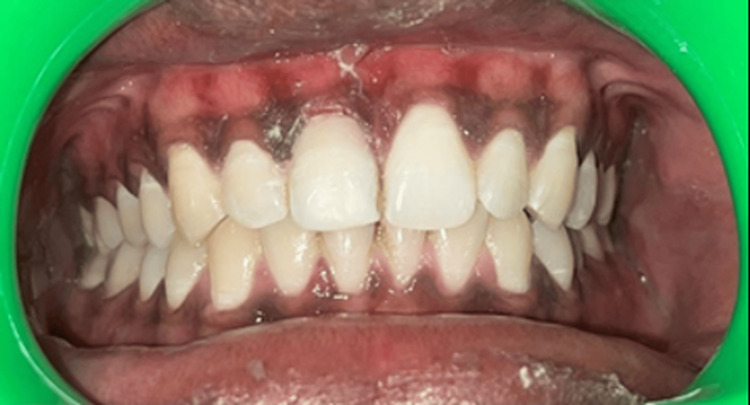
Immediate postoperative image (Case 2)

**Figure 15:**
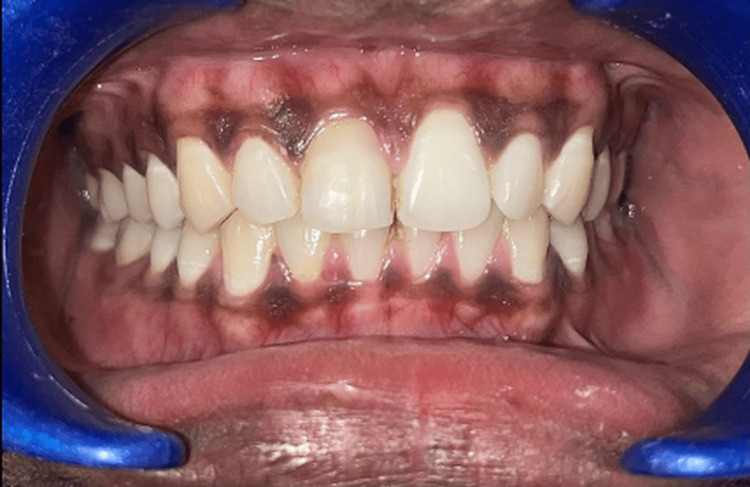
Postoperative image after 12 months (Case 2)

**Figure 16:**
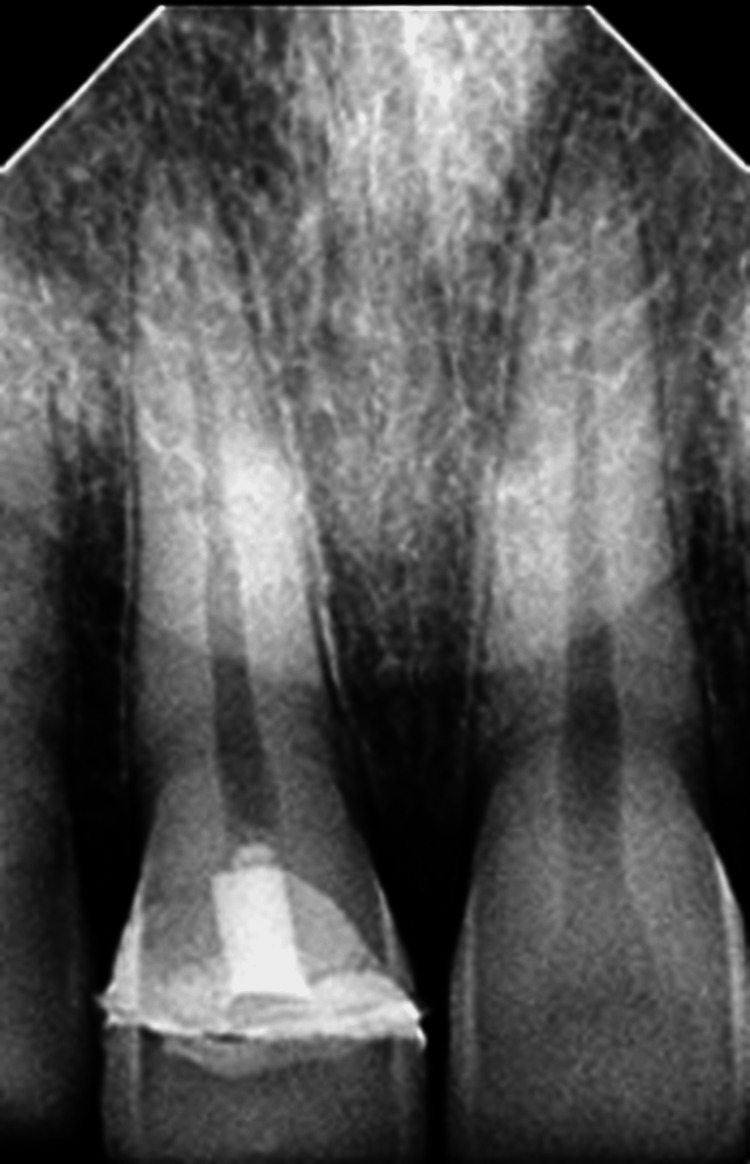
Postoperative radiograph after 12 months showing reparative dentin formation just apical to MTA (Case 2) MTA: mineral trioxide aggregate

**Figure 17:**
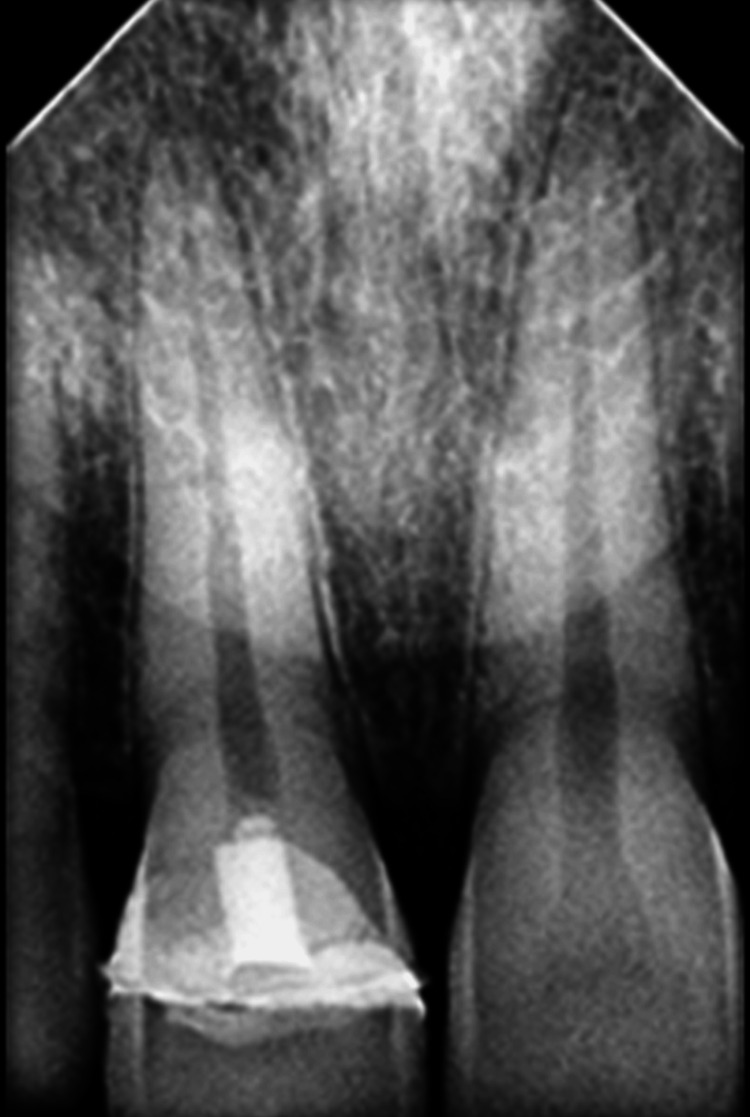
Postoperative radiograph after 24 months showing reparative dentin formation just apical to MTA (Case 2) MTA: mineral trioxide aggregate

Case 3

A 31-year-old patient presented at the Department of Conservative Dentistry and Endodontics with a case of fractured upper anterior teeth. The patient reported a fall two days ago. During the clinical examination, a fracture of Ellis Class III was observed with the fracture line extending subgingivally, and pulp involvement in tooth 11 was seen. Under stimulus, the patient felt pain.

The visible pulp was characterised by a bright red colour, which indicated that the pulp had a well-established blood flow. Both electric pulp testing and thermal testing conducted on the labial surface yielded normal responses from the tooth, suggesting that the pulp was in good health, and also that mobility was absent. Two potential courses of treatment were proposed: either traditional root canal therapy or a partial pulpotomy followed by fragment reattachment using composite resin. As requested by the patient, a partial pulpotomy was carried out. This case involved the elevation of the flap, and isolation was done using a gingival retraction cord that contained an astringent solution of aluminium sulphate. The subsequent procedure was similar to that of the previous cases. Figures 18-25 show a step-by-step procedure.

**Figure 18:**
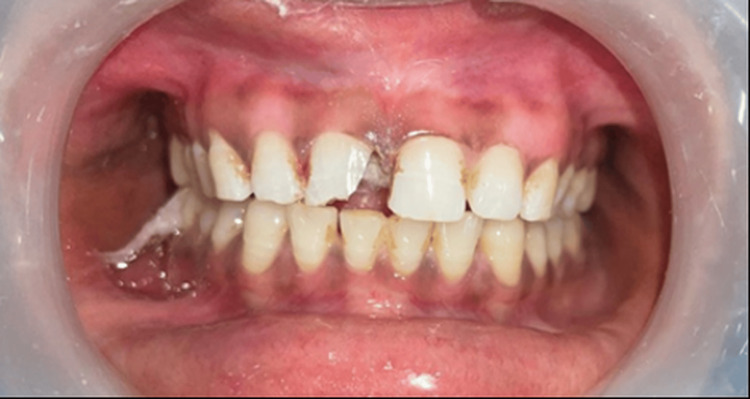
Preoperative image from buccal view showing Ellis class III fracture of 11 (Case 3)

**Figure 19:**
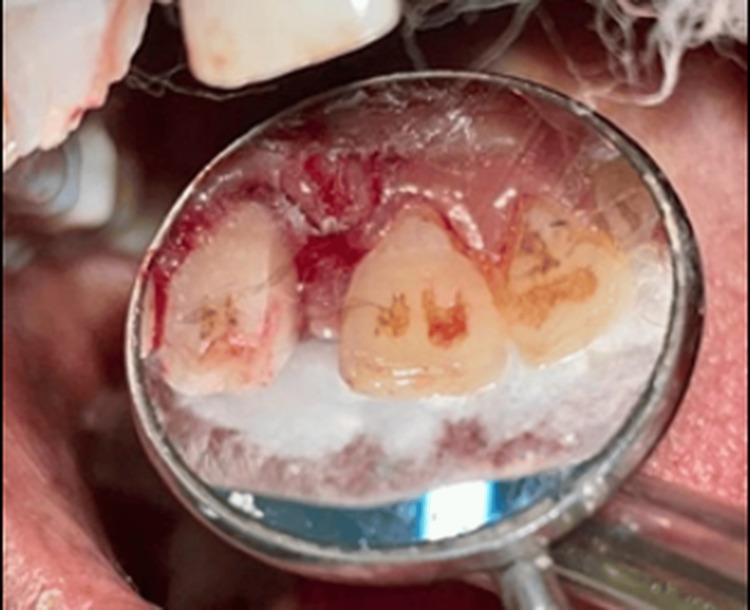
Preoperative image from palatal view showing pulp exposure with 11 (Case 3)

**Figure 20:**
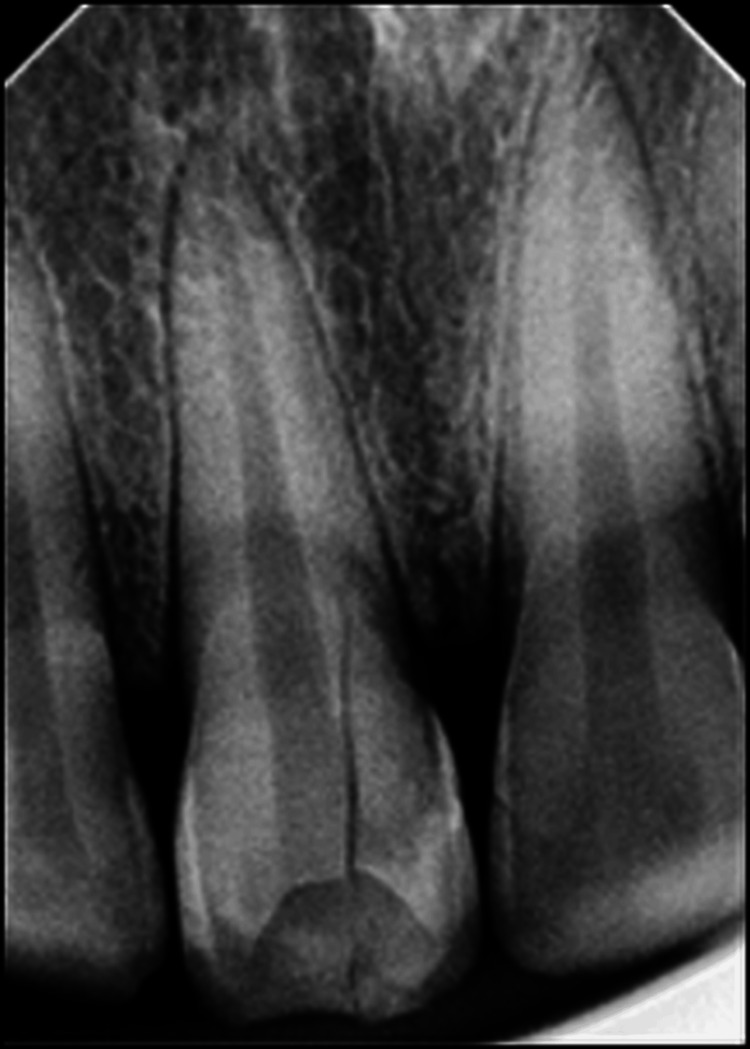
Preoperative radiograph of 11 (Case 3)

**Figure 21:**
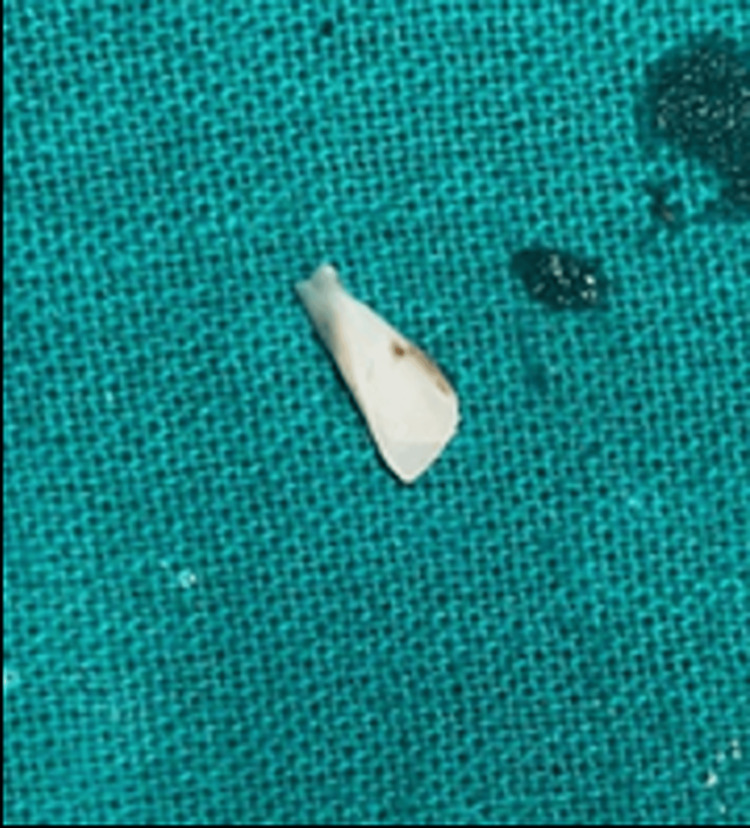
Tooth fragment of 11 (Case 3)

**Figure 22:**
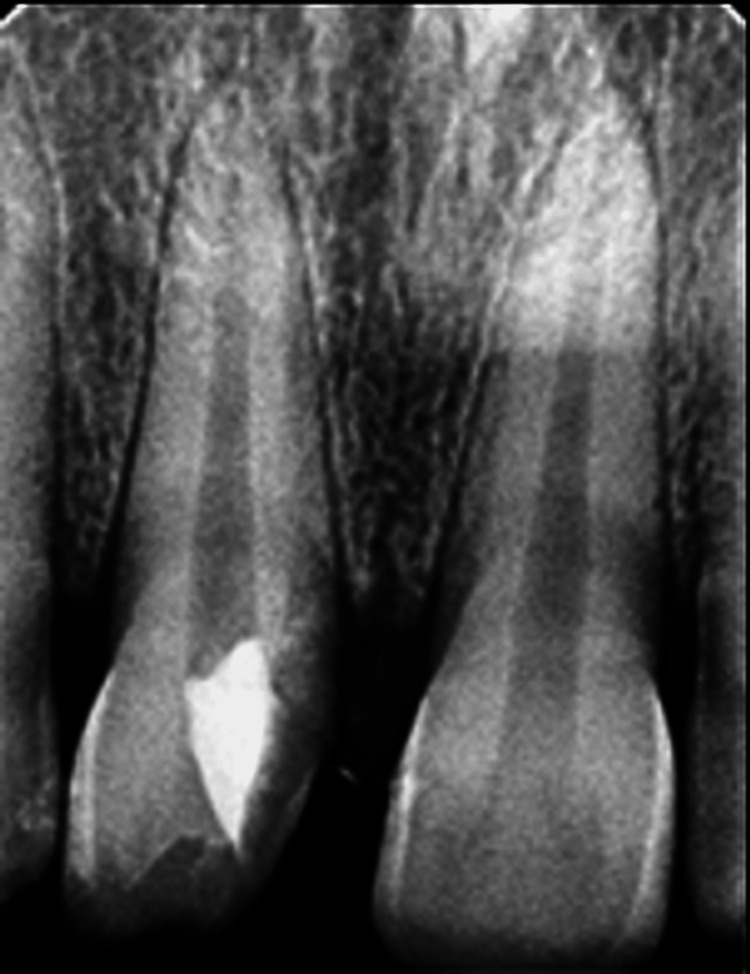
Immediate postoperative image (Case 3)

**Figure 23:**
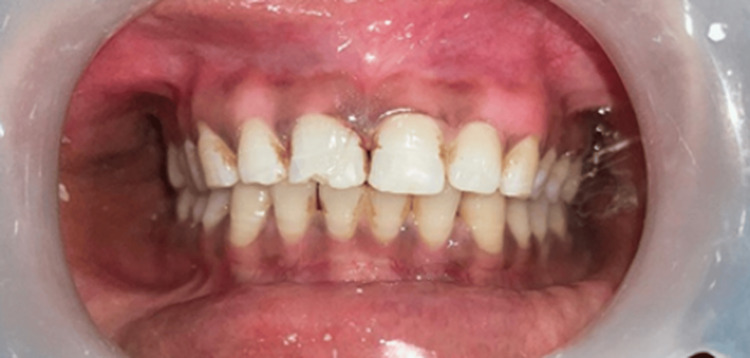
Postoperative image after 12 months (Case 3)

**Figure 24:**
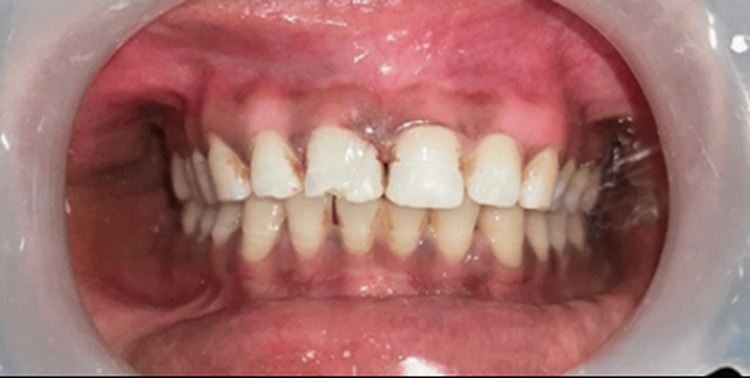
Postoperative image after 12 months (Case 3)

**Figure 25:**
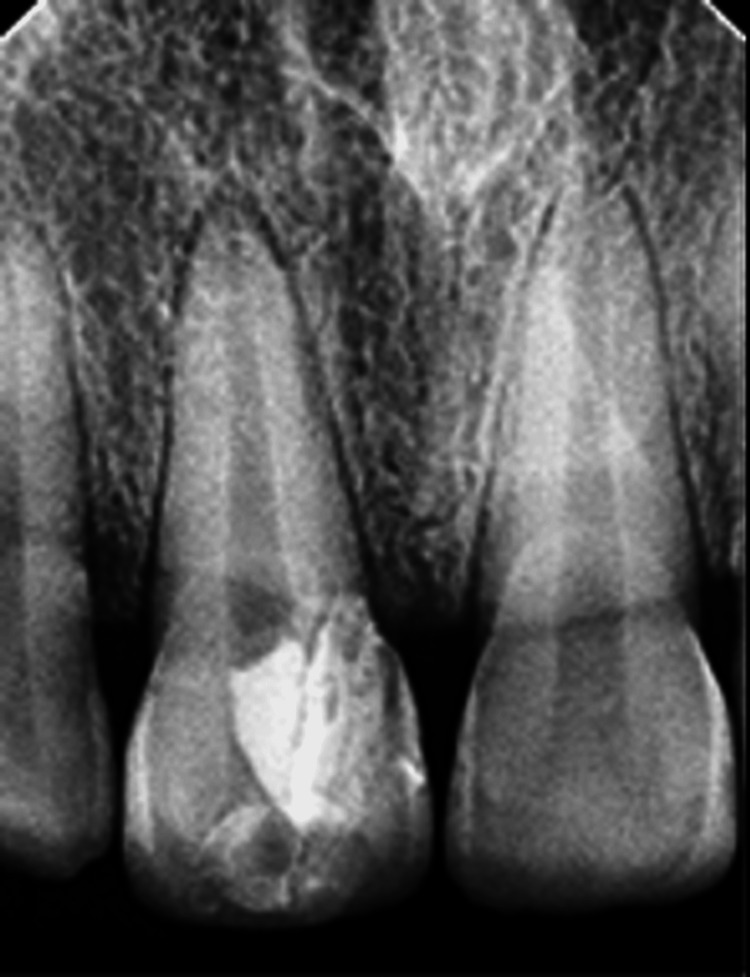
Postoperative radiograph after 12 months showing reparative dentin formation just apical to MTA (Case 3) MTA: mineral trioxide aggregate

Follow-ups

Follow-ups were conducted at 24 hours, one week, 30 days, three months, six months, and 12 months. The subsequent items were verified: Tender to percussion, Electric pulp testing is conducted by positioning the probe in the cervical third of the teeth. Radiographic assessment to identify signs of root resorption or periodontal widening of the ligament space.

Results

Three patients aged 15, 29, and 31 years presented with Ellis Class III fractures involving teeth 11 and/or 21. The time interval between trauma and treatment ranged from 10 hours to 48 hours. The pulp exposure in all cases was categorized as moderate (1-2 mm), with case III demonstrating features of a larger exposure due to subgingival extension.

All cases were treated with partial pulpotomy using MTA, followed by adhesive fragment reattachment. At the 12-month follow-up, all treated teeth were asymptomatic and showed no tenderness to percussion. Electric pulp testing demonstrated a positive vitality response in all treated teeth, and the response was similar to that of the adjacent non-fractured teeth. Radiographic evaluation showed no evidence of periapical pathology or periodontal ligament space widening. A distinct radiopaque layer was observed adjacent to the MTA in all cases, consistent with calcific barrier (dentin bridge) formation.

## Discussion

The prevention or early termination of disease, alongside the preservation of optimal healthy tissue, is a crucial component of any treatment approach and constitutes the foundational principle of minimally invasive dentistry. Pulpotomy is a procedure aimed at preserving the pulp and its functions, including proprioception, innervation, and vascularization. In comparison to root canal treatment (RCT), it is less time-consuming and technically simpler to execute [11]. Also, RCT renders the tooth non-vital, resulting in the removal of a significant portion of tooth structure, which often leads to frequent fractures and eventual tooth loss. Pulpotomy, a vital pulp therapy procedure, involves the excision of the coronal portion of the pulp while preserving the radicular pulp to maintain pulp vitality and can be conducted on teeth without periapical lesions [12].

The efficacy of vital pulp therapy is contingent upon the employed technique, the inflammatory condition of both coronal and radicular pulp, the specific pulp therapy agent utilised, the duration of observation, and the criteria established for assessing success [13]. Histopathologically, a blood clot, fibrin, and inflammatory cells may be observed at the pulp surface immediately following traumatic or carious pulp exposure, resulting from tissue injury and microbial irritation. In these studies, pulpal alterations were defined by a proliferative response consistently linked to superficial inflammation extending no more than 2 mm from the exposure site after seven days [14]. Consequently, partial pulpotomy was conducted in this series [15].

The expression of local biomarkers associated with neutrophil presence in pulp wounds offers a potential means to predict treatment outcomes. The remarkable predictive capability of matrix metalloproteinase 9 (MMP-9; neutrophil gelatinase) concentrations in blood samples obtained from the pulp regarding the success of complete pulpotomy in permanent painful teeth [16]. It was noted that 2.5% NaOCl lavage decreased MMP-9 levels and diminished the postoperative inflammatory response [17].

Age is a critical criterion for the selection of patients for any vital pulp therapy. Older pulps exhibit increased fibrousness, reduced cellularity, and potentially diminished blood supply, thereby influencing treatment outcomes [18].

An outcome indicative of a successful pulpotomy is the formation of a dentin bridge at the amputation site subsequent to the application of calcium hydroxide or silicate cement. As the pH increases, a necrotic zone develops adjacent to the material. A dentine bridge subsequently develops between this layer and the underlying vital pulp. The necrotic tissue ultimately deteriorates and vanishes, creating a gap between the capping material and the bridge. In instances involving materials with a lower pH, such as Dycal, a necrotic zone is similarly established but is resorbed before the dentine bridge forms, which subsequently develops directly against the capping material [19-23]. Dentine bridges created by high pH materials are histologically indistinguishable from those formed by lower pH materials; however, they are more readily identifiable on radiographs due to the gap between the bridge and the calcium hydroxide. Jean et al. discovered that when calcium hydroxide was compared to a mixture of 50% tricalcium phosphate and 50% hydroxyapatite, the latter resulted in a thicker bridge and accelerated formation of normal tubular dentine [24]. The application of chlorhexidine would also mitigate collagen degradation caused by MMP-9 release [25].

Research utilising animal models has demonstrated that the hard tissue bridge formed under calcium hydroxide exhibits numerous imperfections and tunnel defects that may allow for bacterial infiltration, whereas the bridge established following MTA application is devoid of tunnels or flaws. Furthermore, in human teeth, it has been observed that at six months, a dentin bridge measuring 0.43 mm in thickness is evident when using MTA, in contrast to 0.15 mm when no odontoblastic layer is present with calcium hydroxide [26].

The composition of MTA Angelus is tricalcium silicate, dicalcium silicate, bismuth oxide, tricalcium aluminate, calcium oxide, aluminium oxide, and silicon dioxide, while the liquid component consists of distilled water. The initial setting time of White Angelus Mineral Trioxide Aggregate (WAMTA) is approximately 8.5 ± 2.4 minutes [26]. One review article indicated that NaOCl utilised for pulpal lavage and MTA employed as pulp capping material exhibit a higher success rate [27]. MTA possesses the capability to seal dental pulp against bacterial and chemical infiltration and is biocompatible to mitigate toxicity, and tissue irritation can chemically bond to dentin through a diffusion-controlled reaction involving the apatite layer of MTA and the dentin [28].

A case report indicated that recently traumatised teeth with pulp exposure, when treated with partial pulpotomy within 24-48 hours after injury, may serve as a permanent restorative procedure without necessitating endodontic treatment, contingent upon achieving a bacteria-tight seal [18].

A potential source of bias in the study may arise from the fact that all the cases preoperatively demonstrated a positive pulpal response. This could affect the generalizability of the findings, as it excludes cases with negative or variable pulpal responses, potentially skewing the results.

## Conclusions

Within the limitations of these three cases, partial pulpotomy showed favourable clinical outcomes in the management of recently traumatised permanent teeth with pulp exposure. The procedure allowed preservation of pulp vitality while maintaining the structural and functional integrity of the affected teeth. The positive clinical findings observed during follow-up suggest that partial pulpotomy may serve as a conservative and biologically sound alternative to conventional root canal therapy in appropriately selected cases. Further studies with larger sample sizes and longer follow-up periods are required to validate these outcomes and strengthen the available clinical evidence.
